# Economic evaluation of a strategy to shorten the time to surgery with neuraxial anaesthesia compared with usual clinical practice in patients on chronic antiplatelet therapy with a proximal femur fracture

**DOI:** 10.3389/frhs.2024.1423975

**Published:** 2025-01-20

**Authors:** Claudia Erika Delgado-Espinoza, Rosa Maria Antonijoan, Ignasi Gich, Rafael Anaya, Mireia Rodriguez, Angélica Millan, Jordi Llorca, Gemma Usua, Ana Ruiz, Angela Merchán-Galvis, Maria Jose Martinez-Zapata

**Affiliations:** ^1^ Clinical Pharmacology Service, Hospital de la Santa Creu i Sant Pau, Barcelona, Spain; ^2^ Clinical Epidemiology and Public Health Service, Fundació Institut de Recerca Hospital de la Santa Creu i Sant Pau, Barcelona, Spain; ^3^ Centro de Investigación Biomédica en Red de Epidemiología y Salud Pública (CIBER of Epidemiology and Public Health), Madrid, Spain; ^4^ Anaesthesiology Service, Hospital de la Santa Creu i Sant Pau, Barcelona, Spain; ^5^ Orthopedic and Traumatology Surgery Service, Hospital de la Santa Creu i Sant Pau, Barcelona, Spain; ^6^ Anaesthesiology Service, Xarxa Assistencial Universitària de Manresa, Barcelona, Spain; ^7^ Anaesthesiology Service, Hospital de la Vall d’Hebron, Barcelona, Spain; ^8^ Anaesthesiology Service, Hospital Clinic de Barcelona, Barcelona, Spain; ^9^ Departamento de Medicina Social y Salud Familiar, Universidad del Cauca, Popayan, Colombia; ^10^ Public Health and Clinical Epidemiology Service-Iberoamerican Cochrane Centre, Institut de Recerca Sant Pau, Barcelona, Spain

**Keywords:** femur fracture, platelet function test, economic evaluation, randomised clinical trial, neuraxial anaesthesia

## Abstract

**Introduction:**

Before implementing a new health care strategy, it is important to assess effectiveness but also to perform an economic evaluation. The goal of the present study was to perform a comparative economic evaluation of a new strategy aimed at using proposed implementation of the Plateletworks guidance (measurement of platelet function) with usual practice (delayed time to surgery) in patients on chronic antiplatelet treatment and scheduled for surgery with neuraxial anaesthesia due to proximal femur fracture.

**Methods:**

This is an economic evaluation carried out alongside a randomised controlled clinical trial at four centres in Spain. Patients were randomised to undergo either early platelet function-guided surgery (experimental group) or delayed surgery (control group). As AFFEcT trial results demonstrated significative difference between groups in the primary efficacy endpoint, the median time to surgery, a cost-effectiveness analysis was performed. Direct costs associated with hospitalisation until one-month post-discharge were considered and measured from a hospital perspective. All costs were reported in euros. Analyses were performed on a per protocol basis. Effectiveness outcome measures were the incremental cost and incremental cost per reduction in days to surgery. A deterministic sensitivity analysis was implemented to quantify uncertainty.

**Results:**

A total of 156 patients were randomized to the two groups (*n* = 78 per group). A total of 143 patients were included in the per protocol population (75 and 68 patients in the experimental and control groups, respectively). The median time to surgery was 2.30 days (IQR: 1.53–3.73) in the experimental group and 4.87 days (4.36–5.60) in the control group (a reduction of 2.40 days). Total costs during the 1-month study perioperative period were higher in the delayed surgery group (€18,495.19) than for the early surgery group (€16,497.59). The incremental cost was negative (€1,997.60), a statistically significant difference (*P* < 0.05). As measured by the reduction in time (days) to surgery, the incremental cost-effectiveness ratio (ICER) for early surgery was negative (777.28€/day). Sensitivity analysis demonstrated consistent cost saving.

**Conclusion:**

For patients on chronic antiplatelet treatment scheduled to undergo surgery for proximal femur fracture, an individualised strategy guided by a platelet function testing is a cost-saving and cost-effective strategy.

## 1 Introduction

Hip fracture surgery within 24–48 h of admission may be associated with better outcomes (1). In addition, the use of neuraxial anaesthesia in these patients could reduce the length of hospital stay and mortality compared with general anaesthesia (2, 3).

Recently, a randomised controlled trial (the AFFEcT study) conducted by Anaya et al. (4) demonstrated that, compared with usual practice (i.e., delayed surgery), platelet function testing could safely reduce the time to surgery in patients on chronic antiplatelet therapy with a proximal femur fracture. However, the efficiency of this novel strategy has not been evaluated to date.

Although demand for healthcare services continues to grow, available resources are limited. For this reason, for all new treatment strategies, it is important not only to evaluate the efficacy and effectiveness of that strategy, but also to assess its efficiency vs. usual practice (5). In this regard, an economic evaluation can help to determine whether a new approach represents an efficient use of resources or not.

Many studies have been performed to evaluate the impact of the time elapsed between hip fracture and surgery on patient outcomes (6–10). In general, those studies have found that a longer time to surgery is associated with more complications, which is why early surgery is generally recommended. However, relatively few studies have assessed the impact of the time to surgery on economic variables. A retrospective study (11) by Kempenaers et al. found that although delayed surgery had only a modest association with mortality, this delay was associated with a steady increase in healthcare costs. A retrospective study by Chatziravdeli et al. (12) also found that early surgery improves clinical outcomes while also reducing the economic burden. A population-based, propensity-matched cohort study in Canada (13) found that delaying surgery for >24 h after hip fracture was associated with increased medical costs and length of stay.

In this context, the objective of the present study was to evaluate the efficiency of the platelet function-guided early surgery strategy evaluated in the AFFEcT study compared with usual practice (delayed surgery).

## 2 Materials and methods

An economic evaluation was carried out using data collected in a multicentric RCT (AFFEcT study) comparing early platelet function-guided surgery (experimental group) to delayed surgery (control group) in patients with a proximal femur fracture under chronic antiplatelet treatment. In that trial, early surgery was scheduled when functional platelet levels, measured by the Plateletworks assay, were >80 × 109/L.

A health economic analysis plan following per protocol principles was included in the clinical trial protocol. The study inclusion and exclusion criteria, randomisation process, recorded variables, data collection, interventions, and other details have been described in detail elsewhere (14). The Consolidated Health Economic Evaluation Reporting Standards 2022 (CHEERS 2022) Statement was followed (15).

The study was approved by the ethics committee at each participating centre. Patients received detailed written information about the study and were required to provide signed informed consent prior to randomisation.

### 2.1 Population

The study population included patients ≥age 18, of both sexes, with a proximal femur fracture requiring surgery. All patients were receiving antiplatelet agents [cyclooxygenase inhibitors such as acetyl salicylic acid [ASA] or P2Y12 receptor inhibitors [clopidogrel, prasugrel, ticagrelor or ticlopidine]] at the time of admission to the emergency department. The trial was conducted at four hospitals in Spain (Hospital de Santa Creu i Sant Pau, Althaia Xarxa Assistencial Universitària de Manresa, Hospital Vall d'Hebron de Barcelona, Hospital Clínic de Barcelona) between 2017 and 2020.

### 2.2 Sample size

Based on historical data provided by one of the participating centres (Hospital de Santa Creu i Sant Pau), the mean time from admission to surgery in patients who were not taking antiaggregants and without coagulopathies (nor medicated with anticoagulants), was 2.85 [standard deviation (SD), 3.17] days. Based on this SD value (3.17 days) for the time from admission to the intervention, and accepting an alpha risk of 0.05 and a beta risk of <0.2 in a bilateral contrast, a total of 166 participants were needed (78 in each group) to detect a difference ≥1.5 days in the reduction of the time from admission to surgery. A loss rate of 10% was assumed. All calculations were made with the GRANMO calculator, v. 7.10 (June 2010).

### 2.3 Intervention

A total of 156 patients were randomised to undergo either early platelet function-guided surgery (experimental group) or a usual practice control condition (surgery delayed by 3–5 days, depending on the specific antiaggregant treatment).

Plateletworks is an *in vitro* analytic screening test that quantifies the percentage of aggregated or inhibited platelets. Blood samples were collected by venipuncture and results were obtained within 10 min of sampling. Patients randomised to the experimental group who presented a functioning platelet count >80 × 109/L were considered suitable for early surgery under spinal anaesthesia.

Depending on the individual patient, surgery could involve any of the following techniques: osteosynthesis with cannulated screws, dynamic hip screw or intramedullary nail (short or long), or femoral head arthroplasty (monopolar, bipolar, or total). The trial design is described in more detail by Anaya et al. (14)

Patients who did not undergo surgery were excluded from the main analysis. Therefore, the analysis was made on a per protocol basis, which included only randomised patients who underwent surgery.

### 2.4 Outcomes

For the economic evaluation, we considered the direct costs of the following procedures, materials, and outcome measures: length of hospitalisation; diagnostic test (including Plateletworks); surgery costs; prosthesis and osteosynthesis; total blood transfusions; medications; and outpatient hospital visits, re-hospitalisation, rehabilitation, and diagnostic tests performed within 1-month of discharge.

### 2.5 Data collection

Assessments and data collection for the economic evaluation were performed at the following time points: baseline (emergency room), after surgery, at discharge, and at 1-month post-discharge.

### 2.6 Economic evaluation

A within-trial economic evaluation was planned alongside the AFFEcT trial to determine the intervention's efficiency.

AFFEcT trial results demonstrated difference between groups in the primary efficacy endpoint, the median time to surgery (2.30 days in the experimental group and 4.87 days in the control group). On the other hand, all the health outcomes, such as perioperative total blood loss, postoperative complications, perioperative mortality or quality of life were equivalent. As such, and according to a pre-specified economic protocol, a cost-effectiveness analysis was performed.

Analysis took a provider perspective, which considered the costs to the government as a third-party provider of healthcare services in Catalonia. All costs were reported in euros (€) (2020 prices).

The time horizon for the measurement of benefits was 1-month after hospital discharge. This time period was selected because costs incurred more than 1-month after discharge may have been assumed by health care providers other than the surgical hospital.

The analyses were performed on a per protocol basis as patients must have undergone surgery in order to calculate the primary efficacy endpoint.

An incremental cost-effectiveness analysis, undertaken according to a pre-specified economic protocol, measured the net costs and net effectiveness of the intervention compared to usual practice. The incremental cost-effectiveness ratio (ICER) was calculated as the difference in costs between the intervention and usual care divided by the difference in their effect, where the efficacy outcomes were considered to be significantly different between groups (*P* < 0.05). The primary ICER was expressed as the cost per unit reduction in days to surgery.

### 2.7 Costs

Direct healthcare costs incurred from hospital admission to 1-month after discharge were considered in the analysis. Pathway analysis was used to identify the resource items associated with the implementation of the intervention. Resource use was recorded as part of the process evaluation.

Wherever possible, unit costs were obtained from public sources, including state and regional data (16–18). Where public costs were unavailable, local unit costs were obtained from official fees based on calculations from one of the participating centres (Hospital de la Santa Creu I Sant Pau). These costs were assumed to be representative of the costs at the other participating centres.

In both the intervention and control groups, the total costs during hospitalisation comprised the following costs: hospitalisation, diagnostic tests, surgery, and medications. All relevant direct costs during the 1-month-follow up period were considered, including the following costs: rehabilitation, hospitalisation, outpatient visits, and diagnostic or other tests. Other costs, such as those related to imaging tests and emergency care, were not quantified as these are standardised and have no variation between groups.

In the intervention group, the cost of testing with the Plateletworks system was estimated based on data provided by the manufacturer and our own data since there is currently no set fee for this test. This cost was added to the total costs.

No discount rate was applied due to the short time frame (one month) to measure benefits.

### 2.8 Sensitivity analysis

A deterministic sensitivity analysis was undertaken in order to verify the robustness of the results and the critical role of a relevant parameter when key variables or assumptions varied.

We modified the cost of prosthesis or osteosynthesis, assuming the higher cost of these variables, owing to variability of the types of prosthesis or osteosynthesis between centres. Besides, we considered a maximum requirement of blood transfusion and a readmission due to an infectious complication after hospitalisation as part of the sensitivity analysis. All these parameters represent an important component of the costs related to hospitalisation and 1-month after discharge.

Finally, we performed the analysis considering the intention-to-treat population in order to provide an unbiased estimate of the results.

### 2.9 Data analyses

Statistical analyses were performed with the IBM-SPSS statistical software program for Windows, v. 26.0 (IBM-SPSS Inc., Armonk, NY, USA). Data were analysed according to the per protocol principle. Means with SD and mean differences with 95% confidence intervals (CI) were calculated. Differences were assessed using a parametric *t*-test assuming a normal distribution. The level of significance for all statistical analyses was set at 5%.

## 3 Results

A total of 156 patients were randomised and equally allocated to the two groups (*n* = 78 in each group). The per protocol population was 143 (75 and 68 patients in the early and delayed surgery groups, respectively). There were no significant differences between the groups in baseline parameters.

### 3.1 Overall healthcare costs

Table 1 shows detailed overall healthcare costs, including in-hospital costs, considering relevant costs incurred prior to, during, and after surgery until discharge, as well as costs incurred during the 1-month period after hospital discharge. Costs of hospital stay and surgical intervention were the main drivers of the overall healthcare costs.

**Table 1:** Overall healthcare costs (€).

Resource used	Source of unit cost	Total cost	Mean cost/participant ± SD
Pre-surgery, surgery, post-surgery (until discharge) costs			
Hospitalisation costs			
Hospital stay	HSCSP official schedule^a^	1,050,497.28	7,346.13 **±** 3,739.74
Rehabilitation	HSCSP official schedule^a^	6,312.24	44.14 ± 12.78
Diagnostic tests costs			
Platelet determination test	Estimation own sources	7,259.40	50.77 ± 59.82
Laboratory tests	Official journal G. Cat^b^	29,181.70	204.07 ± 35.01
Surgery costs			
Surgical intervention	Official journal G. Cat^b^	1,111,310.11	7,771.40 **±** 566.88
Prosthesis and osteosynthesis	Tender services G. Cat^c^	100,620.14	703.63 ± 488.05
Blood transfusion	HSCSP official schedule^a^	93,537.00	654.10 ± 625.02
Medication costs			
Prophylactic drugs*	SNHH ref. price—BOE^d^	9,091.60	63.58 ± 29.01
Drugs to treat complications/AEs	SNHH ref. price—BOE^d^	4,768.32	39.41 ± 76.69
1-month follow-up			
Rehabilitation	Official journal G. Cat^b^	12,852.00	121.25 ± 91.07
Rehospitalisation	HSCSP official schedule^a^	42,999.00	405.65 **±** 1 296.75
Outpatient visits	Official journal G. Cat^b^	4,750.00	47.03 ± 55.42
Diagnostic, other tests	Official journal G. Cat^b^	7,071.21	112.24 ± 243.17

*Antibiotics and anticoagulants.

^a^ Hospital de la Santa Creu I Sant Pau official schedule.

^b^ Official gazette of the Government of Catalonia (16).

^c^ Tender services of the Government of Catalonia (17).

^d^ Spain National Health System reference prices—Spanish State official gazette (18).

### 3.2 Healthcare costs by groups and incremental costs

Based on the resources consumed during the trial (in-hospital and through the 1-month follow-up period), costs in euros were calculated for both interventions. Costs associated with hospital stay (6,391.91 € vs. 8,398.58 €, *P* = 0.001) and with prophylaxis medication (56.02 € vs. 71.91 €, *P* = 0.001) were significantly lower in the early surgery arm, while costs associated with platelet determination test (96.79 € vs. 0 €, *P* < 0.001 were significantly higher in the early surgery arm. Table 2 shows these results.

**Table 2:** Healthcare costs by study arm (€).

	Early surgery mean costs ± SD (95% CI)	Delayed surgery mean costs ± SD (95% CI)	P*
Pre-surgery, surgery, post-surgery (until discharge) costs			
Hospitalisation costs			
Hospital stay	6,391.91 **±** 3,026.03 (5,695.69–7,088.14)	8,398.58 **±** 4,169.45 (7,389.36–9,407.81)	0.001
Rehabilitation	43.36 **±** 14.01 (40.14–46.58)	45.01 **±** 11.34 (42.26–47.75)	0.443
Diagnostic tests costs			
Platelet determination test	96.79 **±** 48.51 (85.63–107.95)	0.00	<0.001
Laboratory tests	201.25 **±** 36.77 (192.79–209.70)	207.18 **±** 32.97 (199.20–215.16)	0.313
Surgery costs			
Surgical intervention	7,725.82 **±** 559.97 (7,596.98–7,854.66)	7,821.67 **±** 574.33 (7,682.65–7,960.69)	0.314
Prosthesis and osteosynthesis	676.93 **±** 463.44 (570.31–783.56)	733.09 **±** 515.70 (608.26–857.91)	0.777
Blood transfusion	698.41 **±** 677.12 (543.62–854.20)	605.24 **±** 562.97 (468.97–741.51)	0.375
Medication costs			
Prophylaxis	56.02 **±** 28.18 (49.54–62.51)	71.91 **±** 27.79 (65.18–78.64)	0.001
Treatment for complications/AEs	29.07 **±** 47.29 (16.96–41.18)	49.91 **±** 97.29 (24.78–75.04)	0.136
1-month follow-up			
Rehabilitation	119.37 **±** 91.98 (94.96–143.77)	123.43 **±** 90.90 (97.32–149.54)	0.820
Rehospitalisation	528.66 **±** 1,575.57 (114.38–942.93)	552.63 **±** 1 446.66 (137.10–968.16)	0.935
Outpatient visits	53.75 **±** 59.01 (37.95–69.55)	38.67 **±** 49.98 (23.65–53.68)	0.175
Diagnostic/other tests	86.39 **±** 98.26 (21.22–151.55)	151.54 **±** 299.16 (28.05–275.03)	0.302

SD, standard deviation; AE, adverse events.

* *t*-test.

After estimating the total costs, the mean cost associated with early guided surgery was also significantly lower in comparison with delayed surgery (16,497.59 € vs. 18,495.19 €, *P* < 0.05). These cost savings were observed despite acquisition cost for the platelet determination test. Table 3 summarises the total resulting costs for the two study arms.

**Table 3:** Total costs by study arm (€).

	Early guided surgery, *N* = 75 Mean cost ± SD	Delayed surgery, *N* = 68 Mean cost ± SD	Incremental cost (95% CI)	*P* *
Total costs	16,497.59 ± 3,899.21	18,495.19 ± 4,344.63	−1,997.60 (−634.76 to −3,360.43)	<0.05

SD, standard deviation; CI, confidence interval.

* *t*-test.

The incremental cost—calculated as the difference between the two arms (intervention vs. control) in mean total costs—was negative, at €1,997.60. This result indicates a decrease in mean direct costs per patient for the early surgery group vs. the delayed surgery group.

### 3.3 Cost-effectiveness analysis

For the cost-effectiveness analysis, the primary measure of effectiveness was the time (in days) from emergency department admission to surgery in each arm, as reported in the AFFEcT study (4). The median time from admission to surgery was 2.30 days (IQR: 1.53–3.73) in the early surgery group vs. 4.87 days (IQR: 4.36–5.60) in the delayed surgery group (*p* < 0.001), a reduction of 2.57 days. The cost-effectiveness analysis showed that the ICER for early surgery was negative at €777.28€/day (Table 4).

**Table 4:** Cost-effectiveness analysis.

	Early guided surgery *N* = 75	Delayed surgery *N* = 68	Difference
Total costs, €	16,497.59	18,495.19	1,997.60
Median time from admission to surgery, days	2.30	4.87	2.57
ICER	−777.28 €/day^a^		

^a^ Interpreted as a reduction in days to surgery.

ICER, incremental cost-effectiveness ratio.

### 3.4 Sensitivity analysis

A deterministic sensitivity analysis, adjusting for the higher cost of the type of prosthesis or osteosynthesis used in the current practice at the participating centres, and other considering a maximum requirement of blood transfusion in the early guided surgery group and an infectious complication appeared during the first month after discharge in the delayed surgery group, demonstrated a consistent cost saving of 1,941.44€, and 2,879.04€ respectively (*P* < 0.05). Moreover, analysis considering ITT population resulted in a saving of 1,535.99€ (*P* < 0.05), which confirms the robustness of the results. Table 5 outlines these analyses.

**Table 5:** Sensitivity analyses (€).

	Early guided surgery *N* = 75 mean cost ± SD	Delayed surgery *N* = 68 mean cost ± SD	Incremental cost (95% CI)	*P* *
Adjusted by prosthesis or osteosynthesis	17,267.31 ± 3 764.99	19,208.75 ± 4,373.63	−1,941.44 (−595.59 to −3,287.29)	<0.05
Adjusted by blood transfusion and infectious readmission	18,293.50 ± 3 737.30	21,172.54 ± 4,085.88	−2,879.04 (−1,585.74 to −4 172.33)	<0.05
Adjusted by ITT population	15,920.68 ± 4 541.83	17,456.67 ± 4,921.83	−1,535.99 (−38.05 to −3,033.93)	<0.05

SD, standard deviation; CI, confidence interval.

* *t*-test.

## 4 Discussion

In this study, we sought to determine the efficiency of a novel strategy using platelet function testing to reduce the time from femur fracture to surgery in patients on chronic antiplatelet treatment. Total costs were lower in the early surgery group than in the delayed surgery group. Similarly, the incremental cost of early surgery was negative, indicating a significant cost savings. The ICER for early surgery was also negative. These data support the greater efficiency of this novel strategy.

Although the demand for healthcare services continues to grow, the resources available to satisfy this demand are limited. Consequently, one of the main priorities for healthcare providers is to obtain the best value for money spent on health interventions (19).

Economic evaluations are performed to assess the efficiency and allocation of resources to interventions that may improve healthcare quality and health outcomes. These evaluations apply not only to decisions about interventions or services that directly target patients, such as pharmacological treatments and medical devices, but also to decisions about implementation strategies (5).

The AFFEcT RCT concluded that an individualised strategy based on platelet function testing shortens the time to proximal femur fracture surgery under neuraxial anaesthesia in patients on chronic antiplatelet treatment without increasing perioperative adverse events or complications (4). The results of the present economic evaluation, carried out alongside the AFFEcT study, demonstrate that platelet function monitoring is a cost saving and cost-effective strategy from a healthcare provider perspective that could lead to relevant economic impact for the healthcare system. The cost-effectiveness and sensitivity analyses also showed consistent cost savings for the early surgery strategy.

To our knowledge this is the first study to perform an economic evaluation of a strategy designed to guide proximal femur fracture surgery using a platelet function test in patients with chronic antiplatelet treatment.

The hospital stay and surgical intervention were associated with a substantial use of resources, a finding that is consistent with the results of the study by Burgers et al., who evaluated the total medical costs of treating displaced femoral neck fractures with arthroplasty in fit elderly patients (20). In that study, the most important cost category during the first 10 weeks after the fracture was the primary hospital stay, predominantly due to costs related to hospitalisation and index surgery.

In the present study, we found no significant differences between the groups in terms of healthcare costs, except for hospital stay, platelet testing, and prophylactic medications. The fact that costs for the two treatment strategies (i.e., experimental and control) were comparable is congruent with clinical outcomes, which were also similar in the two groups. Notably, the most important factors underlying the reduced costs in the experimental group were a shorter length of hospital stay and, to a lesser extent, a reduction in prophylactic medication. Other studies have reported similar results, finding that delayed surgery is associated with increased medical costs (11–13).

This study has several limitations. First, we did not include the costs associated with outpatient emergency care (i.e., no hospitalisation) and the costs of imaging tests. However, given the similarity between the two study arms in terms of complications rates, these costs were likely similar in the two groups; moreover, it is evident that, within the framework of a comparative analysis, the costs that should be included are those that differ between the alternatives under evaluation (21, 22). In this regard, we believe that omitting the costs associated with outpatient emergency care and imaging tests is justified.

Other aspects of interest to consider when interpreting our results are the characteristics of the healthcare system in Catalonia, and the use of health resources.

Catalonia has a public health system that offers universal healthcare coverage and free access to its inhabitants, therefore the provider and the health system are closely related and contractually full accounted.

About the use of resources, it is important to note that in our context, the hip surgery due to a femur fracture is considered an urgency, not an emergency nor a programmed surgery, regardless of the moment during the hospitalization period in which surgery is performed. Therefore, even if platelet function indicated to perform an early surgery, since this surgery was not considered an emergency, the operating room and other resources could be used as soon as it was possible, but only when they were available without the need for additional resources. For that reason, implementing early surgery did not represent an increase on the utilization of extra resources.

In conclusion, the present within-trial economic evaluation shows that, in patients undergoing surgery for proximal femur fracture receiving chronic antiplatelet treatment, a guided individualised strategy using a platelet function test is cost-effective and can reduce costs, mainly by shortening the time from admission to surgery.

The findings of this study show that, from a healthcare provider's perspective, platelet monitoring has a positive economic impact with no negative influence on clinical outcomes. These data may be of value to decision-makers at the regional or state level considering implementing this novel strategy. Future studies should evaluate this strategy in other geographic and social settings.

## Data Availability

The raw data supporting the conclusions of this article will be made available by the authors, without undue reservation.

## AFFEcT Study Group

Hospital de la Santa Creu i Sant Pau: Rafael Anaya, MD, PhD; Mireia Rodriguez, MD, PhD; José María Gil, MD; Victoria Moral, MD; Angélica Millan, MD; Julio De Caso, MD; Aranzazu González, MD; Noèlia Vilaltahttps://orcid.org/0000-0002-3961-3563, MD; José Mateo https://orcid.org/0000-0002-4227-3256, MD, PhD; Claudia Erika Delgado-Espinoza, MD, PhDc; Rosa María Antonijoan, MD, PhD; Percy-Efrain Pantojahttps://orcid.org/0000-0003-4095-8988, MD, MPH; Angela Merchán-Galvis, MD; Acosta René; Daniela Garrido; Raúl Aguilar; Esther Cánovashttps://orcid.org/0000-0001-7351-9389; Elena Gómez Ibáñez; Maria Jose Martinez-Zapata, MD, PhD. Althaia XAU Manresa: Francesca Reguant, MD, PhD; Jordi Llorca, MD; Joan Camí, MD; Mercé Castejón; José María Huesca, MD; María Sala Grau, MD. Hospital Vall d’Hebron: Patricia Guilaberthttps://orcid.org/0000-0001-6894-2342, MD; Yaiza Garcia-Sanchezhttps://orcid.org/0000-0003-0894-1721, MS; Ernesto Guerra-Farfanhttps://orcid.org/0000-0001-5445-1331, MD; Luis Abarca, MD; Maria Luisa Paños, MD; Jesús Cirbián, MD; Verónica Estepa, MD; Elena Cardona, MD; María Iborra, MD; Maida Navarrete, MD; Jordi Tomas-Hernandez, MD; Jordi Teixidor-Serra, MD; Vicente Molero-Garcia, MD, PhD; Jordi Selga Marsa, MD; Juan Antonio Porcel-Vazquez, MD; Jose Vicente Andres-Peiro, MD. Hospital Clínic: Ana Ruiz, MD; Xavier Sala, MD; Pep Gracia, MD; Lidia Gomez, MD; Pilar Camacho, MD, PhD; Aina Capdevila, MD; Margarita Navarro, MD, PhD; Anna Merino, MD, PhD; Adriana Martinez Camacho, MD; Angel Molina, MD.
